# Ketogenic Diet and Breast Cancer: Recent Findings and Therapeutic Approaches

**DOI:** 10.3390/nu15204357

**Published:** 2023-10-13

**Authors:** Alfio Giuseppe Urzì, Emanuela Tropea, Giuseppe Gattuso, Graziana Spoto, Gabriella Marsala, Daniela Calina, Massimo Libra, Luca Falzone

**Affiliations:** 1 Department of Biomedical and Biotechnological Sciences, University of Catania, 95123 Catania, Italy; alfiourzi20@gmail.com (A.G.U.); peppeg9305@gmail.com (G.G.);; 2 Dipartimento del Farmaco, U.O.C. di Farmaceutica Convenzionata, 95100 Catania, Italy; 3 Department of Clinical Pharmacy, University of Medicine and Pharmacy of Craiova, 200349 Craiova, Romania; 4 Research Center for Prevention, Diagnosis and Treatment of Cancer, University of Catania, 95123 Catania, Italy; 5 Epidemiology and Biostatistics Unit, Istituto Nazionale Tumori IRCCS Fondazione G. Pascale, 80131 Naples, Italy; l.falzone@istitutotumori.na.it

**Keywords:** breast cancer, ketogenic diet, integrated treatments, glycemic load, proteins, ketogenesis

## Abstract

Breast cancer (BC), a complex disease with several influencing factors, is significantly impacted by dietary habits. The ketogenic diet (KD), characterized by high fat and low carbohydrate intake, has gained attention as a potential therapeutic approach, but its effects on BC remain unclear. This review seeks to summarize the current knowledge on the principles of the KD, its metabolic influence on BC cells, and the findings of recent clinical trials, in order to elucidate the potential therapeutic role of the KD in BC management. For these purposes, a comprehensive literature review was conducted selecting preclinical and clinical studies that investigate the relationship between the KD and BC. The selection criteria prioritized studies exploring the KD’s metabolic effects on BC cells and current clinical trials involving the KD in BC management. The reviewed studies provide a diverse range of findings, with some suggesting potential benefits of the KD in inhibiting tumor growth and improving treatment response. However, robust clinical trials providing clear evidence of the KD’s efficacy as a standalone therapeutic approach in BC are still lacking. There are also significant concerns regarding the safety and long-term effects of sustained ketosis in cancer patients. The therapeutic potential of the KD in BC remains an area of active research and debate. While preliminary findings are promising, definitive conclusions are hindered by inconsistent results and limited human trial data. Future research, specifically well-structured, large-scale clinical trials, is necessary to provide a comprehensive understanding of the role of the KD in BC treatment. Until then, caution should be exercised in its application, and patients should continue prioritizing evidence-based, standard-of-care treatments.

## 1. Introduction

Breast cancer (BC) is globally recognized as the second most prevalent form of cancer, and it holds the record as the most frequent type of cancer afflicting women. The rate of BC occurrence is on a steady rise in both advanced and developing nations, largely attributed to the influences of lifestyle choices and environmental hazards [1,2].

Noteworthily, BC is often diagnosed late when the primitive tumor has spread to distant organs like the bone, liver, lung, and brain, forming distant metastases which are often incurable.

Different types of BC, depending on the specific type of tumor cells, have been identified. Adenocarcinomas are the most common BC and include in situ ductal carcinoma (DCIS) and invasive carcinoma where the neoplastic transformation affects gland cells in the milk duct or the lobules. In addition, BC can also be classified according to several molecular features: there are four different molecular types of BC, with specific characteristics that help clinicians determine the most appropriate drug therapy [3].

The most diagnosed form of BC is Luminal A, a tumor that is sensitive to hormones characterized by estrogen (ER+) and progesterone (PR+) receptors and can, therefore, be treated with anti-hormonal therapy as well as classic chemotherapy.

A second type, defined as Luminal B, is positive for hormone receptors (ER+, PR+), but also overexpresses the Her2 (Her2+) receptor, a receptor for epidermal growth factor (EGF) which is associated with cancer aggressiveness.

The third type of BC is exclusively characterized by the over-expression of Her2; therefore, it will be sensitive to a targeted therapy toward this receptor [4].

The last and most difficult-to-treat type is called triple-negative BC, a tumor that is negative for estrogens, progestogens, and Her2 (ER−, PR−, Her2−) receptors. Unfortunately, triple-negative cancer is the one with the fewest therapeutic options [5].

There are several risk factors responsible for the development of BC including sex, aging, estrogen receptor status, family history, and gene mutations. In addition, several studies have demonstrated that daily habits, in particular diet, may be related to an increased risk of BC. In this context, many studies have pointed out the detrimental role of red meat consumption and alcohol abuse [1,6].

To delve deeper, a diet rich in proteins could potentially escalate the risk of BC by boosting the levels of circulating insulin-like growth factor-1 (IGF-1). The link between BC risk and consumption of red meat or proteins is thought to be due to the higher intake of carcinogenic byproducts associated with red meat consumption, as well as an increased intake of hormones derived from the external hormones administered to livestock [7].

As regards alcohol, a majority of studies have demonstrated a proven association between alcohol intake and BC. Acetaldehyde, benzene and n-nitrosodimethylamines, alcohol-carcinogenic byproducts, have been found in alcoholic drinks or as products of alcohol metabolism. Moreover, alcohol determines hormonal alterations causing the suppression of the metabolism of estrogens in the liver, thus increasing the metabolites of circulating estrogens. It also determines the conversion of androgens into estrogens. Alcohol has also been observed to weaken immune system performance, amplify cellular growth, impede the repair of DNA, and foster the invasion and migration of cells [8]. However, the existing epidemiological data on the influence of particular dietary risk factors remains inconclusive largely due to the fact that people’s diet does not comprise isolated foods or nutrients. Instead, people consume a combination of different foods, each with unique constituents that may have a collective impact, by potentially altering biological pathways tied to cancer progression or monitoring. Thus, a more comprehensive assessment of diet, considering all dietary patterns as a whole, is deemed more suitable for deriving valuable insights into the role diet plays in the risk of BC [9]. Indeed, a commitment to wholesome dietary practices has been linked to a significant decrease in BC risk. As such, incorporating nutritional strategies in the treatment plan for BC patients might be viewed as a critical aspect of a comprehensive therapeutic approach. Over recent years, numerous studies have explored the impact of specific diets, such as the Mediterranean diet, on health. This diet, characterized by high consumption of fruits and vegetables, olive oil, fish, and red wine, is known for its high antioxidant content. These antioxidants, including polyphenols, flavonoids, carotenoids, and fibers, coupled with a beneficial profile of fatty acids, are found in the diet’s main components and could potentially lower the risk of BC [9].

Also the ketogenic diet (KD), characterized by high-fat content and low carbohydrate and protein content, has shown potential beneficial effects in BC patients undergoing chemotherapy or in cancer patients in general, due to its composition, which allows the body to pass from a glucose-dependent energy condition (which favors the development and growth of tumors) to an energy condition dependent on fats, where the production of ketone bodies prevents the growth of cancer cells causing the lack of the necessary nourishment [10].

## 2. Review Methodology

A comprehensive literature search was conducted using databases such as PubMed/MedLine, Scopus, Web of Science, Cochrane Library, and Google Scholar. These platforms were selected due to their extensive collection of scholarly articles and research papers. The search was tailored using specific keywords to ensure relevance; these included ((ketogenic diet) AND (breast cancer) AND (therepaeutic approaches))” and“ ((ketogenic diet) AND (breast cancer) AND (research findings))” and similar terms and phrases related to the subject matter.

Inclusion Criteria: Studies that specifically addressed the effects of the KD on BC, focusing on recent findings and therapeutic strategies. Priority was given to recent research articles, randomized controlled trials, cohort studies, case–control studies, and clinical trials, considering both human and animal studies, as long as they were pertinent to the research topic.

Exclusion Criteria: Articles that were not written in English, commentaries, editorials, and those without accessible full text, were excluded. Additionally, studies that did not directly address the influence of the KD on BC were not considered. Although literature reviews were typically excluded, exceptions were made if they provided substantial insights or unique perspectives not found in primary research articles.

The potential articles identified through this process were initially checked for duplication. Titles and abstracts were then screened for suitability, and those that passed this stage underwent a full-text review. The resulting set of articles was thoroughly examined, and their important findings were extracted, summarized, and analyzed within the framework of the KD’s role in BC therapeutics.

## 3. Breast Cancer Epidemiology and Main Clinical-Pathological Features

Based on the most recent epidemiological data related to BC, the year 2020 found approximately 2.3 million new cases. The 5-year prevalence stood at roughly 7,790,717 cases. The age-standardized 5-year relative survival rate for cases diagnosed between 2008 and 2015 in 12 countries in sub-Saharan Africa was reported at 66%. This figure contrasts starkly with the 85–90% survival rate of cases diagnosed in high-income nations between 2010 and 2014. Furthermore, the mortality rate in 2020 was recorded at 6.9%, equivalent to around 690,000 fatalities [11].

BC is characterized by various molecular subtypes, including those expressing the estrogen receptor (ER+), progesterone receptor (PR+), or human epidermal growth factor receptor 2 (HER−2+). Such types can be managed with hormone therapy or Trastuzumab monoclonal antibodies [12]. A distinct subtype, triple-negative breast cancer (TNBC), does not express the estrogen receptor (ER), progesterone receptor (PR), or human epidermal growth factor receptor 2 (HER−2). Clinical traits of TNBC comprise high invasiveness, pronounced metastatic capacity, a predisposition to recur, and a generally unfavorable prognosis. When compared to other BC subtypes, TNBC exhibits a higher level of invasiveness and an elevated rate of recurrence at an early stage. Recurrence typically occurs within 5 years post-surgery and is associated with a particularly poor overall prognosis. Due to the absence of ER, PR, and HER2 expression, TNBC is unresponsive to both endocrine treatment and targeted therapies. The treatment options currently available for TNBC are extremely limited and generally ineffective. Consequently, there is an urgent need for the development of novel therapeutic strategies [5,13].

Notably, BC is a complex, heterogeneous, and multifactorial disease, due to several risk factors. According to the assessment conducted by the International Agency for Research on Cancer (IARC) on pharmaceuticals as human carcinogens, compelling evidence points to the causal relationship between combined estrogen–progestin contraceptives (OCs) and combined estrogen–progestin therapy used during menopause, and the development of BC. Studies indicate that the risk of BC rises with prolonged use of hormone therapy among current users. However, it is still uncertain whether all current preparations and therapies have comparable carcinogenic effects [14]. Night work is increasingly common and a necessity in some sectors of modern society. In particular, night shifts and the lack of daylight lead to the suppression of melatonin production, which has tumor suppressing properties, and disrupt circadian rhythms. All these alterations have been recognized as major mechanisms involved in carcinogenesis [15]. In 2007, the International Agency for Research on Cancer (IARC) categorized night shift work, known to disrupt circadian rhythms, as “probably carcinogenic to humans”. This classification was based on somewhat limited evidence from eight epidemiological studies focusing on BC, reinforced by substantial evidence derived from animal experiments [15].

Genetic changes also play a role in the onset of this tumor. BRCA1 and BRCA2, two tumor suppressor genes, are critical for the homologous recombination repair mechanism of double-strand DNA breaks. Cells with mutated BRCA1 or BRCA2 cannot repair DNA damage via homologous recombination, thus cells use other less efficient mechanisms like the non-homologous end-joining [16]. This may lead to mutations during strand repair and chromosomal rearrangements often occur during consecutive cycles of cell division. Despite the pathogenetic role of gene mutations like BRCA1 and BRCA2 genes, the majority of BC patients do not exhibit clearly identifiable inheritable mutations [17]. In fact, only 5–10% of all BCs are linked to inherited mutations.

Risk factors consistently associated with BC include age, a personal or family history of BC, reproductive factors (i.e., early onset of menstruation, late age at first pregnancy, fewer pregnancies, limited or no breastfeeding, and later menopause), hormonal status and contraceptive or hormone replacement therapy (HRT), alcohol drinking, sedentariness and a lack of exercise, obesity (specifically for postmenopausal BC), and genetic predisposition [18].

HER2 (human epidermal growth factor receptor 2), a membrane tyrosine kinase, influences cell proliferation and survival when activated. The HER2 oncogene, located on chromosome 17q12 [19], is predominantly overexpressed through amplification and is a significant driver of tumor development and progression in a subset of BCs. Around 15% to 20% of BCs exhibit HER2 amplification. Lastly, the most prevalent mutations in ER+ BC are the activating mutations of the PI3 kinase PIK3CA gene, found in approximately 40% of tumors [20].

At present, different therapeutic strategies are available for the treatment of BC. The main pharmacological treatments are summarized below [21]:

### 3.1. Chemotherapy [22,23]

Alkylating Agents: Drugs like Carboplatin (Paraplatin) and Cyclophosphamide function by forming covalent bonds with DNA, thereby interfering with its typical operations. Adverse reactions to these medications can include bone marrow suppression, electrolyte imbalances, and gastrointestinal disturbances such as nausea and vomiting.

Anthracyclines: Medications such as Doxorubicin (Adriamycin) and Epirubicin (Ellence) act by disrupting DNA replication and generating free radicals that cause further harm to cancer cells. Side effects may include both immediate and delayed cardiotoxicity, hair loss (alopecia), myelosuppression, and gastrointestinal disturbances like nausea and vomiting.

Taxanes: Docetaxel (Taxotere) and Paclitaxel (Taxol) are examples of this class of drugs. They inhibit the disassembly of microtubules during mitosis, thereby preventing cell division. Adverse reactions can include hair loss (alopecia), flushing, myelosuppression, gastrointestinal disturbances such as nausea, vomiting, diarrhea, and peripheral neuropathy.

### 3.2. Endocrine Therapy [24]

Aromatase Inhibitors: Medications such as Anastrozole (Arimidex), Exemestane (Aromasin), and Letrozole (Femara) work by blocking the enzyme aromatase, which prevents the transformation of androstenedione into estrone, and testosterone into estradiol. Side effects can include hot flashes, muscle pain (myalgias), and fractures related to osteoporosis.

Selective Estrogen Receptor Modulators: Drugs like Raloxifene (Evista) and Tamoxifen function by competitively binding to estrogen receptors on tumor cells. Adverse reactions can include hot flashes, an elevated risk of thromboembolism, and an increased risk of uterine cancer.

### 3.3. Targeted Therapy with Monoclonal Antibodies

ERBB2-Target Monoclonal Antibodies: Drugs such as Pertuzumab (Perjeta) and Trastuzumab (Herceptin) are monoclonal antibodies that target the extracellular domain of ERBB2, thus preventing the activation of downstream signaling pathways. Adverse reactions can include hair loss (alopecia), fatigue, left heart dysfunction, myelosuppression, and gastrointestinal disturbances such as nausea, vomiting, and diarrhea.

## 4. Breast Cancer and Dietary Factors: Connecting the Dots

Over time, numerous endeavors have been made to determine if, and in what ways, diet may be connected to the onset of BC, its prevention, or even its use as a supplement to various cancer-fighting therapies [25]. With the upward trend in the incidence of BC and the significant surge observed globally, it was theorized that dietary shifts and the emergence of contemporary, ultra-processed foods could play a role in the onset of BC [26]. Specifically, animal-based nutrients and foodstuffs have gained attention as potential contributors to BC, given the apparent significance of obesity and energy balance as factors associated with BC risk.

More recent studies have provided a deeper insight into the influence of dietary factors on specific forms of BC, such as estrogen receptor negative (ER−) BC. Some research suggests that dietary patterns early in life could have a substantial impact on BC development, though the data and evidence are still sparse [27,28]. Predominantly, a sedentary lifestyle combined with poor dietary choices, typified by the overconsumption of hypercaloric foods and underconsumption of healthful foods (rich in ω-3 fatty acids, antioxidants, and fiber), are associated with weight gain and in turn obesity. This status promotes chronic inflammation in adipocytes, establishing a conducive microenvironment for BC emergence and progression. Indeed, obesity has been linked to both an elevated risk of postmenopausal BC and an increase in BC recurrence and mortality [29,30].

In line with the abovementioned data, it was also demonstrated that a hypercaloric diet, characterized by the consumption of red and processed meats, carbohydrates and fatty foods, increases the risk of developing BC due to the increment of the circulating levels of endogenous estrogen, IGF-1, and pro-inflammatory cytokines associated with BC [31]. In contrast, healthy dietary habits characterized by the consumption of fruits and vegetables rich in fiber, ω-3 polyunsaturated fatty acids (PUFAs), and vitamins exert positive effects on human health by reducing chronic inflammation and consequent DNA damage [32].

Several dietary intervention studies on BC patients undergoing chemotherapy have been undertaken to enhance patient outcomes. One of the most notable studies was the Women’s Intervention Nutrition Study (WINS), which included 2437 BC patients with stage I-II tumors and >50 years old. The main aim of the study was the evaluation of the beneficial effects of dietary fat intake reduction on patients’ recurrence-free survival rates. Women treated with dietary interventions showed a fat intake reduction from 29.2% to 20.3%. After five years of follow-up, the patients treated with dietary interventions showed a 24% increment of relapse-free interval compared to the regular diet group [33].

In a 2020 review by Buja and collaborators, they reaffirmed the findings of the third report of the World Cancer Research Fund, which demonstrated the link between the consumption of certain foods with BC characteristics. Specifically, the intake of non-starchy vegetables may decrease the risk of luminal B (ER−) BC, consuming carotenoid-rich foods or adopting diets high in calcium could reduce the risk of BC in both premenopausal and postmenopausal women, as could the consumption of dairy products. The authors also demonstrated that an appropriate intake of vitamins, folates, polyphenols, and other nutrients reduces the risk of BC [34].

The PREDIMED study demonstrated the positive effect of extra-virgin olive oil (EVOO) and a Mediterranean diet in the prevention of BC. Through this study it was possible to establish the anticancer properties of EVOO. Specifically, olive oil represents a rich source of monounsaturated fatty acids, including squalene and oleic acid. Still, EVOO is also rich in polyphenols like oleocanthal, oleuropein, hydroxytyrosol, and lignans, which exert beneficial effects on human health [35]. Studies on cancer cell lines revealed the antiproliferative effect of oleic acid, which mediates the suppression of key oncogenes [36]. Squalene, a hydrocarbon acid, has positive effects in reducing intracellular oxidative stress and DNA oxidative damage in mammary epithelial cells [37]. Polyphenols contained in olive oil might play a role in preventing BC. Oleocanthal has been reported to be able to inhibit cancer cell proliferation, invasion, and migration thus reducing BC progression as demonstrated in vitro and in vivo. Oleuropein is able to increase BC cell apoptosis by stimulating different signal transduction pathways. Finally, hydroxytyrosol is involved in the reduction in intracellular oxidative stress, thus reducing the occurrence of DNA damage as demonstrated in vitro [37,38]. Lignans, which are phytoestrogens, have been associated with a lower risk of BC in postmenopausal women when consumed [39]. Other studies have demonstrated that low-glycemic index diet, and specifically a Mediterranean diet, has several positive effects on patients’ outcome. In this context, the DEDiCa study, based on the administration of a low-glycemic index Mediterranean diet, physical exercise, and vitamin D revealed that diet may improve the prognosis of patients as well as their cardiometabolic parameters and quality of life [40,41,42].

Although the majority of studies to date have focused on the effects of the Mediterranean diet or “healthy diet” as preventive or curative strategies in BC, our understanding of the potential therapeutic effects and benefits of the KD is limited. The following chapters will elucidate key aspects of the KD as well as the primary results attained in approved clinical trials using the KD as a therapeutic intervention in BC.

## 5. Ketogenic Diet

The KD is a high-fat, low-carbohydrate regimen with adequate protein intake. The primary outcome of this diet is ketogenesis, a biochemical process that results in the production of ketones. These ketones are used as an alternative energy source during periods of fasting or more generally when following diets with reduced carbohydrate intake [43].

Of note, our body uses glucose as the first source of energy, but in case of low glucose levels there will be neither the energy necessary for brain function nor efficiency in some biochemical processes such as the tricarboxylic acid cycle (TCA). In this condition, ketone bodies provided the energy necessary for brain functions by crossing the blood–brain barrier. Ketogenesis takes place in the mitochondria of liver cells where fatty acids reach the mitochondria via carnitine palmitoyltransferase, where they are converted into generating acetyl CoA molecules. Subsequently, acetyl CoA is transformed into acetone, acetoacetate, and Beta-Hydroxybutyrate thanks to the action of different enzymes. As already mentioned, the main principle of the KD is a high-fat load and low carbohydrate diet necessary for the synthesis of ketone bodies and a better control of blood glucose levels, which in turn leads to a reduction in the risk of insulin resistance, as well as weight loss [44].

Even if the KD was born as a treatment for refractory epilepsy, today it is under the spotlight as one of the most used diets to lose weight and regulate metabolic disorders [43]. However, thanks to its benefits, numerous studies have shown that the KD can be applied in various fields as a treatment for various pathologies, including neurological disorders, type 2 diabetes, obesity, metabolic syndrome, and different types of cancer [45]. A smaller number of studies have also shown its efficacy in the treatment of polycystic ovarian syndrome and in the regulation of the intestinal microbiota [46,47].

Since its first adoption, various KD protocols have been proposed depending on the type of disease.

The classic KD was first adopted during the 1920s. In the first KD protocol, 90% of the energy comes from fat and 10% from a combination of carbohydrates and proteins. This particular dietary program is achieved by excluding foods high in carbohydrates like fruits and vegetables, bread, pasta, grains, and sugar, and by increasing the consumption of foods high in fats, such as nuts, cream, and butter [48].

A valid alternative is the variant based on medium-chain triglycerides (MCTKD), with a higher proportion of carbohydrates and proteins. Medium-chain triglycerides produce more ketones per kilocalorie of energy than long-chain triglycerides used in the classic KD, require less fat intake to induce ketosis, and are metabolized more rapidly [49,50].

Also, the modified Atkins diet (MAD) has a ketogenic ratio of 0.9:1 (fat:carbs to protein), with approximately 65% of calories derived from fat sources. It consists of the indefinite restriction of carbohydrate intake replaced with fat intake with the primary goal of increasing urinary ketones with respect to the secondary endpoint of weight loss [51,52].

**Figure 1:**
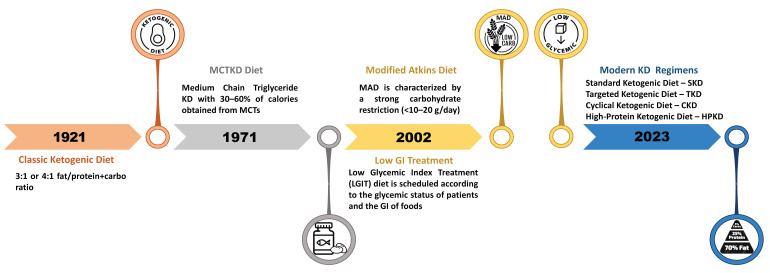
Timeline of the evolution of the ketogenic diet.

Last but not least, the low glycemic index treatment (LGIT) was developed to increase carbohydrate intake, but limits foods with a glycemic index (GI) < 50. In this protocol, patients and their families are initially instructed to exclude high glycemic index carbohydrates from the diet and limit total carbohydrates to 40–60 g/day. The intake of fats and proteins is encouraged. No side effects were noted during the diet. Overall, LGIT produces a low level of ketosis compared to that of the classic KD [53] (Figure 1).

**Figure 2:**
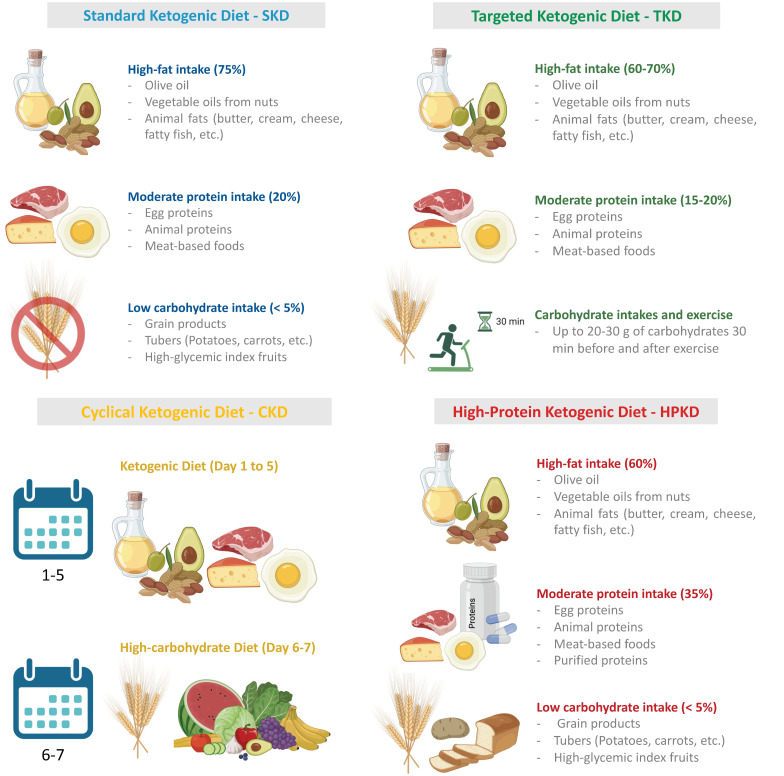
Schematic representation of the most commonly used ketogenic diet regimens.

From a functional point of view, the KD is divided into the standard KD (SKD), which is the currently most adopted form; the targeted ketogenic diet (TKD), most widely used by athletes; the cyclical ketogenic diet (CKD), alternating KD with a carb-based diet and a restricted ketogenic diet for therapeutic uses; or the high-protein KD (HPKD). The SKD is based on a high-fat diet (75%), moderate protein intake (20%), and a limited amount of carbohydrates (5%), with no more than 20–25 g net carbohydrates. The TKD is particularly adopted by athletes who consume high amounts of energy; in this dietary regimen individuals can consume an extra 20–30 g of carbohydrates before and after workouts. The CKD consists of periodical changes in nutritional intake with five days characterized by a KD and 2 days of higher carb refeeds. Finally, the HPKD is a KD regimen mainly based on the consumption of a higher percentage of proteins (30–35%) and the remaining caloric intake obtained from fat (60%) and carbohydrates (5%) [54] (Figure 2).

## 6. Ketogenic Diet in Cancer

Common metabolic traits are observable in most solid tumors, one of which is an elevated glucose intake along with a reliance on glycolysis. Otto Warburg, an early observer of the metabolic distinctions between normal cells and cancerous ones, proposed that cancer cells predominantly employ glycolysis to generate energy, resulting in lactate production, regardless of the availability of oxygen for mitochondrial function [55]. This phenomenon is referred to as the “Warburg Effect”. Subsequent research inferred that this process is connected to cellular proliferation and the expansion of cancer. Thus, in recent years, efforts have been dedicated to identifying a dietary strategy that could serve as an effective supplementary treatment targeting cancer cells [56].

**Figure 3:**
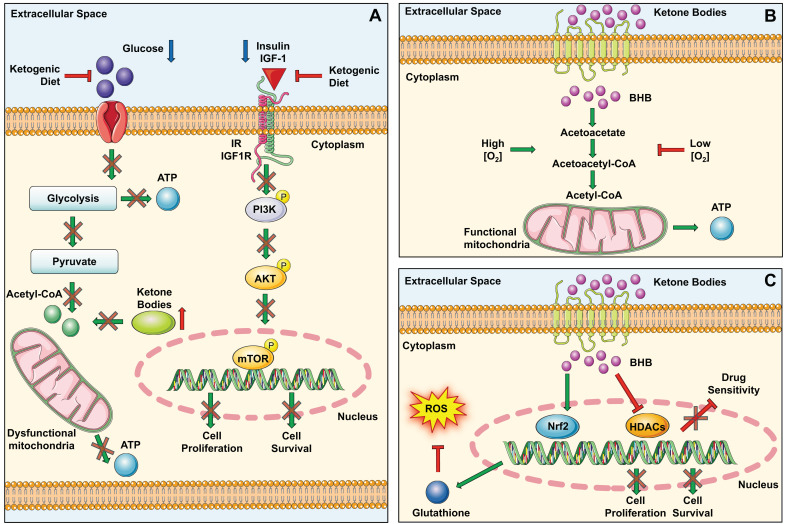
Molecular processes influenced by a ketogenic diet. (**A**) The low carbohydrate intake obtained through a ketogenic diet results in the decrement of glucose, insulin, and IGF-1. As a consequence, tumor-promoting pathways, like the PI3K/Akt pathway, are inhibited, as iss the production of ATP in dysfunctional mitochondria. (**B**) Ketone bodies can be metabolized by cancer cells only in presence of O_2_. In the hypoxic condition typical of tumor cells, BHB cannot be metabolized due to the absence of SCOT, BDH1, and ACAT1. (**C**) BHB favors the gene transcription mediated by Nrf2, with the consequent production of glutathione able to detoxify cells from ROS. In addition, BHB inhibits the effects of several members of the HDAC family, thus inhibiting the proliferation and survival of cancer cells. In parallel, BHB-mediated HDAC-inhibition is responsible for the increase in the cytotoxic effects of chemotherapy on cancer cells.

Many studies have shown that the formulation of a KD could alter the metabolism of cancer cells and in some cases could fight tumor progression. For example, a reduction in carbohydrate intake causes a reduction in circulating blood glucose. This, in turn, causes a reduction in insulin levels and/or IGF receptor signaling pathways, key factors associated with tumor genesis and progression [21] (Figure 3A).

Secondly, the KD also has profound effects on the mitochondrial metabolism of tumor cells. In fact, tumor cells with dysfunctional mitochondria use aerobic fermentation to produce energy. In these cells, the KD induces an increase in ketone bodies and a reduction in glucose levels; in the case of cancer cells with impaired mitochondria, ketone bodies cannot be used for the production of energy and consequently cancer cells cannot grow and survive [57] (Figure 3A). Other studies have been conducted on tumor cells with functional mitochondria, to evaluate whether in this case the KD could act by counteracting its metabolism. Indeed, since these cells are capable of metabolizing ketone bodies only in the presence of certain quantities of oxygen, in hypoxic areas they would not be able to obtain energy from ketone bodies. Of note, the three mitochondrial enzymes involved in the utilization of ketone bodies are SCOT, BDH1, and ACAT1. Therefore, the therapeutic efficacy of the KD should also be evaluated based on the expression of these enzymes (Figure 3B) [58].

Another typical feature of tumor cells is the increase in the amount of reactive oxygen species (ROS), driven by the instability associated with the microenvironment and mitochondrial dysfunction. The increase in ROS can result in resistance to therapeutic treatments and in tumor progression [59]. Specifically contributing to the balancing of ROS levels are the antioxidant potential of glutathione and the activity of the transcription factor Nrf2. Some preclinical studies in mice treated with a KD have shown an increase in glutathione levels associated with the activation of the Nrf2 factor, a transcription factor that regulates the gene expression of a large variety of antioxidant and detoxifying cytoprotective enzymes (Figure 3C) [60,61]. Such protective effects exerted by the KD may be useful for the protection of different tumors with an etiology based on environmental or occupational risk factors which promote oxidative stress including, lung cancer, skin tumors, mesothelioma, brain tumors, etc. [59,62,63,64,65].

The KD might play a significant role in the modulation of gene expression. A key feature of the KD is the induction of ketogenesis, resulting in an increased production of beta-hydroxybutyrate (BHB)—the most abundant ketone body. BHB is implicated in the suppression of histone deacetylases (HDACs), which are enzymes that participate in gene transcription by removing acetyl groups from lysine residues on histones. The correlation between HDACs and various cancer states has been thoroughly substantiated. Multiple studies have explored BHB’s inhibitory impact on these enzymes, leading to consequent gene regulation. Key findings by Shimazu et al., along with others, affirm that BHB impairs the functioning of HDAC1, HDAC3, and HDAC4, thus underlining the physiological significance of this mechanism [66,67]. Emerging evidence suggests that a KD possesses potential anticancer properties, such as inhibiting tumor growth, shielding normal cells from chemotherapy or radiation damage, intensifying the toxic effects of chemotherapy on cancer cells, and mitigating inflammation (Figure 3C). The KD, in comparison to standard anticancer medications and therapies, is cost-effective, user-friendly, and generally well-received. An increasing number of preclinical studies indicate that the KD, as a dietary intervention, could be a powerful anticancer strategy. The bulk of these preclinical investigations reveals that the KD curtails tumor growth, prolongs survival, delays tumor onset, and reverses cancer-induced cachexia [56,58,68].

All these molecular and cellular effects driven by the KD result in the dysregulation of key signal transduction pathways associated with BC development and progression including the PI3K/Akt, the mTOR, and MAPK signal transduction pathways [10]. It was also postulated that a KD and other dietary regiments influence the behavior of BC cells by modulating the expression levels of microRNAs and the functionality of the vitamin D/VDR pathway [69].

## 7. Current Clinical Studies on Ketogenic Diet and Breast Cancer

Starting from the interesting results obtained in vitro and in preclinical studies, several research groups have proposed a KD within clinical trials aimed at patients with BC. More specifically, key metrics considered in the evaluation of KD efficacy are the impact of diet on body weight, the reduction in recurrence, the reduction in hyperglycemia the better response to different treatments, including letrozole, paclitaxel, alpelisib, etc. as showed in the clinical studies reported in Table 1.

### 7.1. Effects of Ketogenic Diet in Overweight and Obese Women with Breast Cancer (NCT05234502—Ongoing)

In this clinical study, Alkin SB and team introduce a KD protocol for overweight and obese women with BC, who are due to undergo neoadjuvant chemotherapy. The study aims to enroll 56 BC patients and randomize them into two arms: one group will follow the KD protocol alongside standard neoadjuvant therapy (including an anthracycline and/or taxane) for 12 weeks, while the second group will adhere to a balanced and healthy diet in conjunction with the standard neoadjuvant therapy for the same duration. The study plans to document a range of clinical-pathological features including tumor size, nutritional status, biochemical findings, anthropometric data, quality of life, sensory and motor neuropathy, and survival rates. Post the 12-week neoadjuvant therapy, the effects of the KD on prognosis and the previously listed factors will be compared. Secondary endpoints include the potential impacts of the KD on chemotherapy-induced neuropathy and pathological responses. Additionally, an evaluation of the overall quality of life of patients following the proposed treatments will be carried out. As hypothesized by the study’s initiators, a KD may enhance patients’ body composition, mitigate obesity-related complications, and reduce neuropathy, potentially leading to decreased pharmaceutical usage and hospital visits.

The findings from this research aim to enhance the health and quality of life of women living with BC.

### 7.2. A 2-Week Ketogenic Diet in Combination with Letrozole to Modulate PI3K Signaling in ER+ Breast Cancer (NCT03962647—Ongoing)

Rexer and colleagues proposed a clinical trial based on a neoadjuvant treatment to determine the tolerability and effects of a 2-week protocol with a very low carbohydrate KD in combination with letrozole for patients with early-stage operable ER+ BC. Besides this fundamental primary endpoint, other objectives of the study will be the evaluation of cancer cell proliferation, measured by analyzing Ki67 expression, in patients treated or not with a KD, the evaluation of the activity of the insulin and PI3K pathways, the measurement of changes in weight, body composition, and insulin resistance as well as the occurrence of a ketogenic state when a KD is administered concomitantly to endocrine therapy.

This protocol is currently ongoing and is aimed at 36 BC patients as conceived as a pilot and feasibility study. Specifically, each patient will be evaluated at baseline to measure metabolic parameters. Patients will be randomized into two groups treated with a daily dose of 2.5 mg letrozole with or without a 2-week KD. A biopsy from the surgically removed tumor will be conducted to assess cell proliferation, with these results then compared to the pre-treatment diagnostic biopsy data.

### 7.3. Ketogenic Diet and Chemotherapy in Affecting Recurrence in Patients with Stage IV Breast Cancer (KETO-CARE) (NCT03535701—Completed)

In this preliminary study, the researchers enlisted 20 individuals diagnosed with Stage IV BC, with the goal of assessing the potential for a KD to enhance the effectiveness of chemotherapy in mitigating the recurrence of BC. The principal objective was to evaluate the feasibility of inducing nutritional ketosis through a KD in women about to commence palliative chemotherapy for advanced-stage BC, in addition to assessing the diet’s impact on tumor progression and health biomarkers. To facilitate this, patients were divided into two groups: the first group underwent standard treatment with the drug paclitaxel, while the second group was given standard paclitaxel treatment along with a regimented KD, prepared in the research kitchen, for a duration of three months. Commencing two weeks before the end of the controlled feeding period, patients also began a three-month free-living KD program. This program consisted of group-based sessions, individual consultations, and online digital content designed to educate patients on incorporating a ketogenic dietary pattern into their everyday lifestyle. Although the study was completed, no results were reported in clinicaltrial.gov.

### 7.4. Preventing High Blood Sugar in People Being Treated for Metastatic Breast Cancer (NCT05090358—Ongoing)

Iyengar and colleagues have proposed a randomized clinical trial involving 106 patients with metastatic BC. These patients will be categorized into three distinct experimental groups. The first group consists of postmenopausal patients with HR-positive, HER2-negative, metastatic BC with PIK3CA mutation treated at least with a single line of endocrine-based therapy at the diagnosis of metastatic disease. This group will receive standard of care (SOC) endocrine therapy (fulvestrant) and PI3K inhibition (alpelisib) plus a ketogenic dietary schedule. The second group, similar in composition to the first, will be administered a low carbohydrate diet along with the SOC endocrine therapy and PI3K inhibition. The third group will receive treatment involving SGLT2i therapy, again in combination with the SOC endocrine therapy and PI3K inhibition.

The primary goal of this study is to evaluate the reduction in hyperglycemia and the prevalence of grade 3/4 hyperglycemia-free rate at the 12-week mark. Ultimately, the study aims to investigate the potential beneficial effects a KD with low-carbohydrate intake as well as the effects of canagliflozin in preventing glycemia and enhancing the effectiveness of cancer treatment in PIK3CA-mutant BC patients treated with targeted therapy (alpelisib) and endocrine therapy (fulvestrant).

### 7.5. Diet Modification in Patients with Luminal Early Breast Cancer Candidate for Primary Surgery (MACS) (NCT04469296—Ongoing)

In this clinical trial, D’Hondt and colleagues proposed dietary protocol aimed at 75 BC patients before BC surgery, divided into three different treatment groups: the first group is composed of control patients without dietary interventions; the second group is composed of patients enrolled in the “ketogenic arm” treated with iso-caloric KD characterized by a scheduled intake of low glycemic index foods and carbohydrate-free foods ad libitum. The patients will be followed by a nutritionist who give dietary advice to increase the consumption of lipid foods for a total of 65% of energy intake. In addition, a list of unauthorized sugars and sweetener substitutes will be proposed; the third group is composed of patients enrolled in the “protein-restricted diet” group where the patients are treated with a KD with a 20% reduction in protein compared to the “ketogenic diet” group. The main aim of this clinical trial is to examine the practicability of dietary modifications—specifically, adherence to either a ketogenic or protein-restricted diet—for patients diagnosed with early luminal BC over a period of 9 ± 1 day prior to undergoing BC surgery.

As secondary endpoints, metabolic changes and potential antitumor effects will be evaluated following the specific treatment in each arm of the study in order to observe biological modifications supporting the beneficial effects of KD in BC. Finally, patients’ quality of life, beliefs, anxiety and depression on the compliance of such treatments will also be analyzed.

### 7.6. Ketogenic or LOGI Diet in a Breast Cancer Rehabilitation Intervention (KOLIBRI) (NCT02092753—Completed)

The purpose of this clinical trial was to evaluate the feasibility, safety, and tolerability of a KD and low glycemic and insulinemic (LOGI) diet in comparison to standard dietary regimens. Moreover, the study aimed to evaluate the enhancement of patient’s quality of life and physical performance during the rehabilitation phase for BC patients. The trial was an open-label study involving 150 BC patients, monitored for a total duration of 20 weeks divided into three different stages: three weeks of in-house protocol, 16 weeks of out-of-house step, and a final week of in-house intervention.

The primary goal of this KOLIBRI study was to analyze the impacts of a high-fat KD or a moderate-fat LOGI diet on the quality of life and physical performance of patients during rehabilitation. Specifically, the patients underwent a KD that consisted of 75% of daily caloric intake from fat consumption (particularly from vegetable fats derived from plant oils, nuts, avocados, and animal fats from butter, cream, cheese, eggs, fatty fish, etc.), very low carbohydrates (around 20–30 g/day), and balanced protein intake (1.4 g/kg body weight/day).

The LOGI diet involved up to 120 g of carbohydrates (preferably from vegetables and fruits), high protein (1.7 g/kg body weight/day), and the remaining calories from fats. For the control group, patients adhered to the standard recommendations of the German Society for Nutrition (DGE). Although the study has been completed, no results were reported on the clinicaltrial.gov portal. However, the researchers published an article demonstrating how low-carb and KDs enhance the quality of life, physical performance, body composition, and metabolic health of patients with BC [70].

### 7.7. Comparison of Healthy Diets on Breast Cancer Markers (KetoBreast) (NCT02744079—Completed)

Fine and colleagues have proposed a study investigating the impacts of a KD on patients with BC. This pilot trial includes 65 patients with either ER+ or ER− BC, who have undergone a post-breast mass biopsy. The participants are divided into two distinct arms: one group adheres to a ketogenic insulin-inhibiting diet, while the second group follows a low-fat diet incorporating whole grains, fruits, and vegetables.

The primary objective of the study is to contrast the impacts of these two diets on ER-positive BC tissues. The diets are administered in the interim period between diagnosis and surgical removal. Additionally, proliferation (as measured by Ki-67) and apoptosis (as assessed by TUNEL) rates are evaluated in conjunction with the pathology of the surgical specimen, to compare changes in biomarkers.

## 8. Clinical Limitations and Pitfalls in the Application of the Ketogenic Diet to Breast Cancer

One of the key limitations of the KD is its strict regimen requiring high fat, moderate protein, and minimal carbohydrate intake. This dietary shift can be challenging for many patients, which might lead to inconsistent adherence and subsequently affect the clinical outcomes [66]. A significant pitfall of the KD is the potential for essential nutrient deficiencies. The diet’s restriction on certain food groups could result in inadequate intake of key vitamins and minerals essential for overall health. The long-term adverse effects of KD consumption are related to unwanted metabolic changes, including elevated blood lipid levels, which may escalate the risk of cardiovascular diseases. Other common and sometimes severe side effects are hepatic steatosis due to hyperlipidemia, kidney stones due to the formation of protein aggregates, hypoproteinemia as a consequence of the excessive protein metabolism and co-enzyme deficiencies [71]. Also, the response to the KD can vary significantly among individuals due to differences in metabolic responses and tumor characteristics. This individual variability presents a considerable clinical limitation, as it hampers the predictability of treatment response [71,72].

Other authors have questioned the safety of KD administration in cancer patients due to the high intake of proteins of animal origin. As already mentioned in the Introduction section, a diet rich in animal proteins increases the risk of BC due to the activation of signal transduction pathways which increase cell proliferation and cancer aggressiveness [7,73]. In this context, some authors have proposed plant-based dietary regimens to be associated with a KD in order to reduce the protein intake derived from animal sources [74].

The absence of standardized KD protocols for BC patients is another significant clinical gap. This lack of uniformity hampers the comparability of results across studies and consistency in clinical application. The majority of studies on the KD have focused on early-stage cancers or used the diet in conjunction with other therapies and its efficacy in the context of advanced or metastatic BC is still under-explored, thus limiting its scope of application.

These clinical limitations and pitfalls underscore the necessity for further research and the development of comprehensive protocols to enhance the efficacy and applicability of the KD in BC management.

## 9. Conclusions and Future Perspectives

Different studies have highlighted the potential therapeutic role of the KD in the management of BC patients. For instance, in their review article Jemal M and colleagues (2021) report the positive effects of a KD on BC by describing the molecular effects determined by a KD in tumor cells [10]. Other authors have investigated the beneficial properties of a KD related to the response to chemotherapy [75]. Starting from these observations, in our review of the recent literature, we want to update the current knowledge of the molecular process driven by the KD in BC as well as to provide different examples of KD regimens that can be adopted as supportive treatments in BC patients. In addition, to the best of our knowledge, this review is the first which collects nutritional, molecular, and clinical findings related to the KD in BC by presenting all the clinical studies where a KD was adopted as a treatment strategy.

Overall, the data contained in our review revealed that several preclinical studies have shown that the KD can reduce tumor growth, enhance the sensitivity of BC cells to chemotherapy, and reduce cancer-related symptoms. The KD, a high-fat, low-carbohydrate dietary regimen, shifts the body’s metabolism from being glucose-based to being ketone-based. This metabolic shift appears to generate metabolic stress on cancer cells due to their reliance on glycolysis for energy. Furthermore, the KD has been found to modulate inflammation and oxidative stress, which are critical processes in cancer progression. However, clinical studies evaluating the effects of the KD in BC patients are limited, and results have been mixed. Some studies report potential benefits in terms of tumor growth control, overall survival, and quality of life (see Table 1), while others show no significant difference when compared to standard care [76].

There is a clear need for more robust, well-designed clinical trials to conclusively determine the efficacy of the KD as a therapeutic approach for BC. These trials should aim to evaluate not only the diet’s impact on tumor progression but also its effects on patient wellbeing, quality of life, and potential side effects. It will also be critical to understand which patient populations are most likely to benefit from this approach. Genomic and metabolic profiling of tumors might provide valuable information about which patients would respond favorably to a KD, enabling precision medicine approaches. The KD’s effects should be evaluated in conjunction with existing treatment modalities, such as chemotherapy, radiation, and targeted therapies. Additionally, investigating the mechanisms of action through which the diet exerts its effects on cancer cells will provide valuable insight and could lead to the development of novel therapeutic strategies. Finally, developing guidelines for implementing the KD in a clinical setting will be crucial. This will include strategies for dealing with common challenges of the diet, such as maintaining patient adherence, managing potential side effects, and integrating the diet with existing therapeutic regimens.

In conclusion, the findings here reported strongly support the potential beneficial effects of a KD for BC, which may counteract some tumor-promoting pathways by reducing glucose, insulin, and IGF-1 levels as well as inhibiting the detrimental effects of ROS. Therefore, the KD holds promise as an additional treatment for BC patients; however, more clinical studies are needed to clearly establish the duration of treatment and its potential benefits.

## Figures and Tables

**Table 1:** Clinical trials using the KD in breast cancer patients registered at https://clinicaltrials.gov (accessed on 20 July 2023).

ClinicalsTrials Identifier	Status	Objective of the Clinical Trial	Intervention	Disease
NCT05234502	O	Impacts of KD on Overweight and Obese Women Diagnosed with Breast Cancer	Alternative 1: Comprehensive and Well-Balanced Diet Regimen (Carbohydrates: 45–60%, Protein: 10–20%, Fat: 20–35%) Alternative 2: Ketogenic Diet Regimen (Carbohydrates: 6%, Protein: 19%, Fat: 75%)	Breast Cancer Female
NCT03962647	O	Two-Week Ketogenic Diet Coupled with Letrozole for the Regulation of PI3K Signaling in ER+ Breast Cancer	Supplemental Diet: Two-Week Ketogenic Diet Regimen Pharmaceutical Treatment: Letrozole	Estrogen Receptor-positive Breast Cancer
NCT03535701	C	Exploring the Role of Ketogenic Diet and Chemotherapy in Modulating Recurrence in Stage IV Breast Cancer Patients	Nutritional Addendum: Dietary Intervention Strategy Alternate: Analysis of Lab-Based Biomarkers Pharmaceutical: Administration of Paclitaxel	Stage IV Breast Cancer AJCC v6 and v7
NCT05090358	O	Mitigating Hyperglycemia in Patients Undergoing Treatment for Metastatic Breast Cancer	Nutritional Supplement: Implementation of the KD Nutritional Supplement: Adoption of a Low-Carbohydrate Diet Pharmaceutical: Usage of Alpelisib	Breast Cancer Breast Cancer Stage IV Metastatic Breast Cancer
NCT04469296	O	Adjusting Nutritional Intake in Luminal Early Stage Breast Cancer Patients Candidate for Primary Surgery	Nutritional Intervention 1: Iso-Caloric KD Regimen Nutritional Intervention 2: Protein-Limited Dietary Approach	Breast Cancer Surgery
NCT02092753	C	Exploring Ketogenic or LOGI Dietary Approaches in Breast Cancer Recovery Intervention (KOLIBRI Study)	Alternate Approach: Standard Dietary Plan (SD) Experimental Approach 1: Ketogenic Dietary Regimen (KD) Experimental Approach 2: Low Glycemic and Insulinemic Diet (LOGI)	Quality of Life
NCT02744079	C	Comparative Study of Healthful Dietary Patterns on Biomarkers of Breast Cancer	Behavioral Modification 1: Adoption of a Low-Carbohydrate Diet Behavioral Modification 2: Implementation of a Low-Fat Diet	Breast Neoplasms

Abbreviations: C, closed; O, ongoing. A more detailed description of the studies reported in Table 1 is provided below to better elucidate the main results obtained by using the KD in patients with BC.

## Data Availability

The data reported in the present manuscript are available at https://pubmed.ncbi.nlm.nih.gov (accessed on 26 July 2023) and at https://clinicaltrials.gov (accessed on 20 July 2023).

## References

[1] Łukasiewicz S., Czeczelewski M., Forma A., Baj J., Sitarz R., Stanisławek A. (2021). Breast Cancer-Epidemiology, Risk Factors, Classification, Prognostic Markers, and Current Treatment Strategies-An Updated Review. Cancers.

[2] Falzone L., Grimaldi M., Celentano E., Augustin L.S.A., Libra M. (2020). Identification of Modulated MicroRNAs Associated with Breast Cancer, Diet, and Physical Activity. Cancers.

[3] Eliyatkın N., Yalçın E., Zengel B., Aktaş S., Vardar E. (2015). Molecular Classification of Breast Carcinoma: From Traditional, Old-Fashioned Way to A New Age, and A New Way. J. Breast Health.

[4] Lin K., Baritaki S., Vivarelli S., Falzone L., Scalisi A., Libra M., Bonavida B. (2022). The Breast Cancer Protooncogenes HER2, BRCA1 and BRCA2 and Their Regulation by the iNOS/NOS2 Axis. Antioxidants.

[5] Maqbool M., Bekele F., Fekadu G. (2022). Treatment Strategies Against Triple-Negative Breast Cancer: An Updated Review. Breast Cancer.

[6] Liu H., Shi S., Gao J., Guo J., Li M., Wang L. (2022). Analysis of risk factors associated with breast cancer in women: A systematic review and meta-analysis. Transl. Cancer Res..

[7] Lo J.J., Park Y.M., Sinha R., Sandler D.P. (2020). Association between meat consumption and risk of breast cancer: Findings from the Sister Study. Int. J. Cancer.

[8] Sun Q., Xie W., Wang Y., Chong F., Song M., Li T., Xu L., Song C. (2020). Alcohol Consumption by Beverage Type and Risk of Breast Cancer: A Dose-Response Meta-Analysis of Prospective Cohort Studies. Alcohol Alcohol..

[9] Wang X., Liu X., Jia Z., Zhang Y., Wang S., Zhang H. (2021). Evaluation of the Effects of Different Dietary Patterns on Breast Cancer: Monitoring Circulating Tumor Cells. Foods.

[10] Jemal M., Molla T.S., Asmamaw Dejenie T. (2021). Ketogenic Diets and their Therapeutic Potential on Breast Cancer: A Systemic Review. Cancer Manag. Res..

[11] Sung H., Ferlay J., Siegel R.L., Laversanne M., Soerjomataram I., Jemal A., Bray F. (2021). Global Cancer Statistics 2020: GLOBOCAN Estimates of Incidence and Mortality Worldwide for 36 Cancers in 185 Countries. CA Cancer J. Clin..

[12] Onitilo A.A., Engel J.M., Greenlee R.T., Mukesh B.N. (2009). Breast cancer subtypes based on ER/PR and Her2 expression: Comparison of clinicopathologic features and survival. Clin. Med. Res..

[13] Yin L., Dceruan J.J., Bian X.W., Yu S.C. (2020). Triple-negative breast cancer molecular subtyping and treatment progress. Breast Cancer Res..

[14] Di Sibio A., Abriata G., Forman D., Sierra M.S. (2016). Female breast cancer in Central and South America. Cancer Epidemiol..

[15] Hansen J. (2017). Night Shift Work and Risk of Breast Cancer. Curr. Environ. Health Rep..

[16] Lavoro A., Scalisi A., Candido S., Zanghì G.N., Rizzo R., Gattuso G., Caruso G., Libra M., Falzone L. (2022). Identification of the most common BRCA alterations through analysis of germline mutation databases: Is droplet digital PCR an additional strategy for the assessment of such alterations in breast and ovarian cancer families?. Int. J. Oncol..

[17] Narod S.A., Salmena L. (2011). BRCA1 and BRCA2 mutations and breast cancer. Discov. Med..

[18] Kashyap D., Pal D., Sharma R., Garg V.K., Goel N., Koundal D., Zaguia A., Koundal S., Belay A. (2022). Global Increase in Breast Cancer Incidence: Risk Factors and Preventive Measures. BioMed Res. Int..

[19] Krishnamurti U., Silverman J.F. (2014). HER2 in breast cancer: A review and update. Adv. Anat. Pathol..

[20] Litton J.K., Burstein H.J., Turner N.C. (2019). Molecular Testing in Breast Cancer. Am. Soc. Clin. Oncol. Educ. Book.

[21] Trayes K.P., Cokenakes S.E.H. (2021). Breast Cancer Treatment. Am. Fam. Physician.

[22] Schmidt M. (2014). Chemotherapy in early breast cancer: When, how and which one?. Breast Care.

[23] Falzone L., Bordonaro R., Libra M. (2023). SnapShot: Cancer Chemotherapy. Cell.

[24] Nabieva N., Fasching P.A. (2021). Endocrine Treatment for Breast Cancer Patients Revisited-History, Standard of Care, and Possibilities of Improvement. Cancers.

[25] Shapira N. (2017). The potential contribution of dietary factors to breast cancer prevention. Eur. J. Cancer Prev..

[26] Romieu I., Khandpur N., Katsikari A., Biessy C., Torres-Mejía G., Ángeles-Llerenas A., Alvarado-Cabrero I., Sánchez G.I., Maldonado M.E., Porras C. (2022). Consumption of industrial processed foods and risk of premenopausal breast cancer among Latin American women: The PRECAMA study. BMJ Nutr. Prev. Health.

[27] Lee J.E. (2021). Diet Before and After Breast Cancer. Adv. Exp. Med. Biol..

[28] Lillycrop K.A., Burdge G.C. (2014). Breast cancer and the importance of early life nutrition. Cancer Treat. Res..

[29] Pang Y., Wei Y., Kartsonaki C. (2022). Associations of adiposity and weight change with recurrence and survival in breast cancer patients: A systematic review and meta-analysis. Breast Cancer.

[30] Riondino S., Formica V., Valenzi E., Morelli C., Flaminio V., Portarena I., Torino F., Roselli M. (2023). Obesity and Breast Cancer: Interaction or Interference with the Response to Therapy?. Curr. Oncol..

[31] Klement R.J., Fink M.K. (2016). Dietary and pharmacological modification of the insulin/IGF-1 system: Exploiting the full repertoire against cancer. Oncogenesis.

[32] Farvid M.S., Barnett J.B., Spence N.D. (2021). Fruit and vegetable consumption and incident breast cancer: A systematic review and meta-analysis of prospective studies. Br. J. Cancer.

[33] Parry B.M., Milne J.M., Yadegarfar G., Rainsbury R.M. (2011). Dramatic dietary fat reduction is feasible for breast cancer patients: Results of the randomised study, WINS (UK)—Stage 1. Eur. J. Surg. Oncol..

[34] Buja A., Pierbon M., Lago L., Grotto G., Baldo V. (2020). Breast Cancer Primary Prevention and Diet: An Umbrella Review. Int. J. Environ. Res. Public Health.

[35] Schwingshackl L., Hoffmann G. (2014). Monounsaturated fatty acids, olive oil and health status: A systematic review and meta-analysis of cohort studies. Lipids Health Dis..

[36] Carrillo C., Cavia Mdel M., Alonso-Torre S.R. (2012). Antitumor effect of oleic acid; mechanisms of action: A review. Nutr. Hosp..

[37] Sánchez-Quesada C., Gutiérrez-Santiago F., Rodríguez-García C., Gaforio J.J. (2022). Synergistic Effect of Squalene and Hydroxytyrosol on Highly Invasive MDA-MB-231 Breast Cancer Cells. Nutrients.

[38] Mattioli A.V., Serra F., Spatafora F., Toni S., Farinetti A., Gelmini R. (2022). Polyphenols, Olive oil and Colonrectal cancer: The effect of Mediterranean Diet in the prevention. Acta Biomed..

[39] Toledo E., Salas-Salvadó J., Donat-Vargas C., Buil-Cosiales P., Estruch R., Ros E., Corella D., Fitó M., Hu F.B., Arós F. (2015). Mediterranean Diet and Invasive Breast Cancer Risk Among Women at High Cardiovascular Risk in the PREDIMED Trial: A Randomized Clinical Trial. JAMA Intern. Med..

[40] Vitale S., Palumbo E., Polesel J., Hebert J.R., Shivappa N., Montagnese C., Porciello G., Calabrese I., Luongo A., Prete M. (2023). One-year nutrition counselling in the context of a Mediterranean diet reduced the dietary inflammatory index in women with breast cancer: A role for the dietary glycemic index. Food Funct..

[41] Montagnese C., Porciello G., Vitale S., Palumbo E., Crispo A., Grimaldi M., Calabrese I., Pica R., Prete M., Falzone L. (2020). Quality of Life in Women Diagnosed with Breast Cancer after a 12-Month Treatment of Lifestyle Modifications. Nutrients.

[42] Porciello G., Montagnese C., Crispo A., Grimaldi M., Libra M., Vitale S., Palumbo E., Pica R., Calabrese I., Cubisino S. (2020). Mediterranean diet and quality of life in women treated for breast cancer: A baseline analysis of DEDiCa multicentre trial. PLoS ONE.

[43] Sampaio L.P. (2016). Ketogenic diet for epilepsy treatment. Arq. Neuropsiquiatr..

[44] McPherson P.A., McEneny J. (2012). The biochemistry of ketogenesis and its role in weight management, neurological disease and oxidative stress. J. Physiol. Biochem..

[45] Zhu H., Bi D., Zhang Y., Kong C., Du J., Wu X., Wei Q., Qin H. (2022). Ketogenic diet for human diseases: The underlying mechanisms and potential for clinical implementations. Signal Transduct. Target. Ther..

[46] Paoli A., Mancin L., Bianco A., Thomas E., Mota J.F., Piccini F. (2019). Ketogenic Diet and Microbiota: Friends or Enemies?. Genes.

[47] Paoli A., Mancin L., Giacona M.C., Bianco A., Caprio M. (2020). Effects of a ketogenic diet in overweight women with polycystic ovary syndrome. J. Transl. Med..

[48] Wheless J.W. (2008). History of the ketogenic diet. Epilepsia.

[49] Shcherbakova K., Schwarz A., Apryatin S., Karpenko M., Trofimov A. (2022). Supplementation of Regular Diet with Medium-Chain Triglycerides for Procognitive Effects: A Narrative Review. Front. Nutr..

[50] Watanabe S., Tsujino S. (2022). Applications of Medium-Chain Triglycerides in Foods. Front. Nutr..

[51] Zilberter T., Zilberter Y. (2018). Ketogenic Ratio Determines Metabolic Effects of Macronutrients and Prevents Interpretive Bias. Front. Nutr..

[52] Gauthier A., Simic N., Jones K.C., RamachandranNair R. (2019). Modified Atkins Diet with slow reduction of carbohydrate. Epilepsy Behav. Rep..

[53] Sondhi V., Agarwala A., Pandey R.M., Chakrabarty B., Jauhari P., Lodha R., Toteja G.S., Sharma S., Paul V.K., Kossoff E. (2020). Efficacy of Ketogenic Diet, Modified Atkins Diet, and Low Glycemic Index Therapy Diet Among Children with Drug-Resistant Epilepsy: A Randomized Clinical Trial. JAMA Pediatr..

[54] Shilpa J., Mohan V. (2018). Ketogenic diets: Boon or bane?. Indian J. Med. Res..

[55] Warburg O., Wind F., Negelein E. (1927). The metabolism of Tumors in the body. J. Gen. Physiol..

[56] Talib W.H., Mahmod A.I., Kamal A., Rashid H.M., Alashqar A.M.D., Khater S., Jamal D., Waly M. (2021). Ketogenic Diet in Cancer Prevention and Therapy: Molecular Targets and Therapeutic Opportunities. Curr. Issues Mol. Biol..

[57] Vidali S., Aminzadeh S., Lambert B., Rutherford T., Sperl W., Kofler B., Feichtinger R.G. (2015). Mitochondria: The ketogenic diet--A metabolism-based therapy. Int. J. Biochem. Cell Biol..

[58] Weber D.D., Aminzadeh-Gohari S., Tulipan J., Catalano L., Feichtinger R.G., Kofler B. (2020). Ketogenic diet in the treatment of cancer—Where do we stand?. Mol. Metab..

[59] Genc S., Pennisi M., Yeni Y., Yildirim S., Gattuso G., Altinoz M.A., Taghizadehghalehjoughi A., Bolat I., Tsatsakis A., Hacımüftüoğlu A. (2022). Potential Neurotoxic Effects of Glioblastoma-Derived Exosomes in Primary Cultures of Cerebellar Neurons via Oxidant Stress and Glutathione Depletion. Antioxidants.

[60] Milder J.B., Liang L.P., Patel M. (2010). Acute oxidative stress and systemic Nrf2 activation by the ketogenic diet. Neurobiol. Dis..

[61] Pinto A., Bonucci A., Maggi E., Corsi M., Businaro R. (2018). Anti-Oxidant and Anti-Inflammatory Activity of Ketogenic Diet: New Perspectives for Neuroprotection in Alzheimer’s Disease. Antioxidants.

[62] Loreto C., Caltabiano R., Graziano A.C.E., Castorina S., Lombardo C., Filetti V., Vitale E., Rapisarda G., Cardile V., Ledda C. (2020). Defense and protection mechanisms in lung exposed to asbestiform fiber: The role of macrophage migration inhibitory factor and heme oxygenase-1. Eur. J. Histochem..

[63] Loreto C., Lombardo C., Caltabiano R., Ledda C., Hagnas M., Filetti V., Rapisarda V. (2020). An In vivo Immunohistochemical Study on MacroH2A.1 in Lung and Lymph-Node Tissues Exposed to an Asbestiform Fiber. Curr. Mol. Med..

[64] Giambò F., Leone G.M., Gattuso G., Rizzo R., Cosentino A., Cinà D., Teodoro M., Costa C., Tsatsakis A., Fenga C. (2021). Genetic and Epigenetic Alterations Induced by Pesticide Exposure: Integrated Analysis of Gene Expression, microRNA Expression, and DNA Methylation Datasets. Int. J. Environ. Res. Public Health.

[65] Nikolouzakis T.K., Falzone L., Lasithiotakis K., Krüger-Krasagakis S., Kalogeraki A., Sifaki M., Spandidos D.A., Chrysos E., Tsatsakis A., Tsiaoussis J. (2020). Current and Future Trends in Molecular Biomarkers for Diagnostic, Prognostic, and Predictive Purposes in Non-Melanoma Skin Cancer. J. Clin. Med..

[66] Huang C., Wang P., Xu X., Zhang Y., Gong Y., Hu W., Gao M., Wu Y., Ling Y., Zhao X. (2018). The ketone body metabolite β-hydroxybutyrate induces an antidepression-associated ramification of microglia via HDACs inhibition-triggered Akt-small RhoGTPase activation. Glia.

[67] Shimazu T., Hirschey M.D., Newman J., He W., Shirakawa K., Le Moan N., Grueter C.A., Lim H., Saunders L.R., Stevens R.D. (2013). Suppression of oxidative stress by β-hydroxybutyrate, an endogenous histone deacetylase inhibitor. Science.

[68] Cortez N.E., Mackenzie G.G. (2021). Ketogenic Diets in Pancreatic Cancer and Associated Cachexia: Cellular Mechanisms and Clinical Perspectives. Nutrients.

[69] Falzone L., Gattuso G., Candido S., Tomaselli A., Fagone S., Spandidos D.A., Libra M. (2023). Vitamin D and microRNAs: Role in the pathogenesis and prognosis of breast cancer. Int. J. Epigenet..

[70] Kämmerer U., Klement R.J., Joos F.T., Sütterlin M., Reuss-Borst M. (2021). Low Carb and Ketogenic Diets Increase Quality of Life, Physical Performance, Body Composition, and Metabolic Health of Women with Breast Cancer. Nutrients.

[71] Batch J.T., Lamsal S.P., Adkins M., Sultan S., Ramirez M.N. (2020). Advantages and Disadvantages of the Ketogenic Diet: A Review Article. Cureus.

[72] Crosby L., Davis B., Joshi S., Jardine M., Paul J., Neola M., Barnard N.D. (2021). Ketogenic Diets and Chronic Disease: Weighing the Benefits Against the Risks. Front. Nutr..

[73] Dierssen-Sotos T., Gómez-Acebo I., Gutiérrez-Ruiz N., Aragonés N., Amiano P., Molina de la Torre A.J., Guevara M., Alonso-Molero J., Obon-Santacana M., Fernández-Tardón G. (2020). Dietary Constituents: Relationship with Breast Cancer Prognostic (MCC-SPAIN Follow-Up). Int. J. Environ. Res. Public Health.

[74] Shah U.A., Iyengar N.M. (2022). Plant-Based and Ketogenic Diets as Diverging Paths to Address Cancer: A Review. JAMA Oncol..

[75] Plotti F., Terranova C., Luvero D., Bartolone M., Messina G., Feole L., Cianci S., Scaletta G., Marchetti C., Di Donato V. (2020). Diet and Chemotherapy: The Effects of Fasting and Ketogenic Diet on Cancer Treatment. Chemotherapy.

[76] Klement R.J., Champ C.E., Kämmerer U., Koebrunner P.S., Krage K., Schäfer G., Weigel M., Sweeney R.A. (2020). Impact of a ketogenic diet intervention during radiotherapy on body composition: III-final results of the KETOCOMP study for breast cancer patients. Breast Cancer Res..

